# Overview of Radiolabeled Somatostatin Analogs for Cancer Imaging and Therapy

**DOI:** 10.3390/molecules25174012

**Published:** 2020-09-02

**Authors:** Romain Eychenne, Christelle Bouvry, Mickael Bourgeois, Pascal Loyer, Eric Benoist, Nicolas Lepareur

**Affiliations:** 1 UPS, CNRS, SPCMIB (Laboratoire de Synthèse et Physico-Chimie de Molécules d’Intérêt Biologique)—UMR 5068, Université de Toulouse, F-31062 Toulouse, France; eychenne@arronax-nantes.fr (R.E.); benoist@chimie.ups-tlse.fr (E.B.); 2 Groupement d’Intérêt Public ARRONAX, 1 Rue Aronnax, F-44817 Saint Herblain, France; mickael.bourgeois@univ-nantes.fr; 3 CNRS, CRCINA (Centre de Recherche en Cancérologie et Immunologie Nantes—Angers)—UMR 1232, ERL 6001, Inserm, Université de Nantes, F-44000 Nantes, France; 4 Comprehensive Cancer Center Eugène Marquis, Rennes, F-35000, France; c.bouvry@rennes.unicancer.fr; 5 CNRS, ISCR (Institut des Sciences Chimiques de Rennes)—UMR 6226, Univ Rennes, F-35000 Rennes, France; 6 INRAE, Institut NUMECAN (Nutrition, Métabolismes et Cancer)—UMR_A 1341, UMR_S 1241, Inserm, Univ Rennes, F-35000 Rennes, France; pascal.loyer@univ-rennes1.fr

**Keywords:** somatostatin analogs, radiolabeling, radiopharmaceuticals, radionuclide therapy, imaging

## Abstract

Identified in 1973, somatostatin (SST) is a cyclic hormone peptide with a short biological half-life. Somatostatin receptors (SSTRs) are widely expressed in the whole body, with five subtypes described. The interaction between SST and its receptors leads to the internalization of the ligand–receptor complex and triggers different cellular signaling pathways. Interestingly, the expression of SSTRs is significantly enhanced in many solid tumors, especially gastro-entero-pancreatic neuroendocrine tumors (GEP-NET). Thus, somatostatin analogs (SSAs) have been developed to improve the stability of the endogenous ligand and so extend its half-life. Radiolabeled analogs have been developed with several radioelements such as indium-111, technetium-99 m, and recently gallium-68, fluorine-18, and copper-64, to visualize the distribution of receptor overexpression in tumors. Internal metabolic radiotherapy is also used as a therapeutic strategy (e.g., using yttrium-90, lutetium-177, and actinium-225). With some radiopharmaceuticals now used in clinical practice, somatostatin analogs developed for imaging and therapy are an example of the concept of personalized medicine with a theranostic approach. Here, we review the development of these analogs, from the well-established and authorized ones to the most recently developed radiotracers, which have better pharmacokinetic properties and demonstrate increased efficacy and safety, as well as the search for new clinical indications.

## 1. Introduction

Somatostatin (SST), also called somatotropin release inhibiting factor (SRIF), is a cyclic peptide hormone, first isolated in 1968 from an ovine hypothalamus, and actually identified in 1973 [1]. It was originally discovered as a growth hormone inhibitor, but is now known to be involved in the inhibition of numerous metabolic processes relating to neurotransmitters, endocrine secretions (e.g., growth hormone, insulin, glucagon, and gastrin) but also modulating exocrine secretions (e.g., gastric acid and pancreatic enzymes). In the body, its synthesis takes place in the form of an inactive precursor of 116 amino acids (AA), preprosomatostatin, which is then converted by the action of proteases into prosomatostatin (96 AA). Depending on where it is produced in the body, enzymes do not cleave the pro-peptide on the same amino acid motif, resulting in two distinct active forms, SRIF-28 and SRIF-14. Although SRIF-14 is predominant in the central nervous system and SRIF-28 in the digestive tract, the distribution of these two biologically active forms is similar.

In the early 1990s, concomitantly to studies on the binding properties and mechanisms of action of somatostatin, five receptor subtypes were discovered (SSTR1 to SSTR5) [2]. These subtypes belong to the family of receptors coupled to G-proteins, and their length varies from 364 to 418 AA. They all exhibit seven α helices with transmembrane domains and most of the differences between subtypes are found in the extracellular (N-terminal) and intracellular (C-terminal) ends. SSTR-1, -3, -4, and -5 have a single subtype, while two variants exist for SSTR2, called SSTR2A and SSTR2B. SSTR1 to 4 link SRIF-14 and -28 with a very high affinity (in the nanomolar order), whereas SSTR5 shows an affinity 5 to 10 times higher, but for SRIF-28 only.

**Figure 1:**
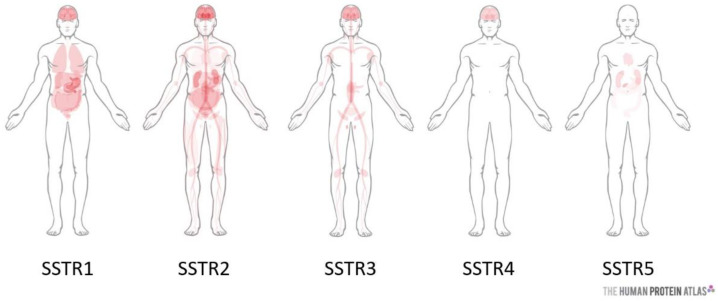
Somatostatin receptors (SSTRs) biodistribution in the body (from The Human Protein Atlas https://www.proteinatlas.org/).

Somatostatin receptors are widely distributed in healthy tissues, with distinct expression throughout the body (Figure 1). It is quite possible to find several subtypes in the same tissue. Each of the SSTRs is involved in the regulation of the various processes: (i) SSTR1 is involved in the antisecretory effects of growth hormone, prolactin (a peptide hormone involved in lactation, reproduction, growth, and immunity) and calcitonin (regulation of calcemia); (ii) SSTR2 also inhibits the secretion of growth hormone and adrenocorticotropin (hormone that stimulates the adrenal glands), glucagon, insulin, interferon-γ (protein produced by immune cells), and stomach acid; (iii) SSTR5 has the same inhibiting effect on growth hormone, adrenocorticotropin, insulin, and inhibits the secretion of amylase (digestive enzyme constituting saliva and pancreatic juice); (iv) SSTR3 reduces cell proliferation and causes cell apoptosis; (v) the functions of SSTR4 are not yet well defined [3].

**Figure 2:**
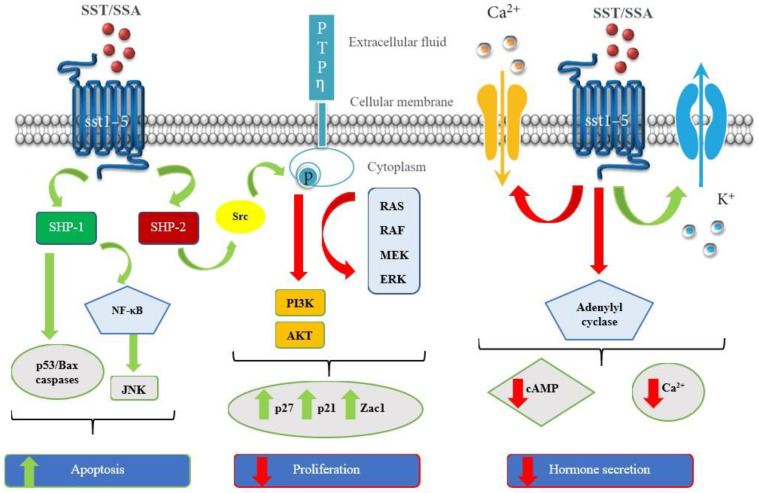
Schematic representation of the signaling pathways induced by somatostatin receptors activation. Green arrows: activated pathways; red arrows: inhibited pathways. Adapted from [8].

The effects of somatostatin are expressed through different signaling pathways [4,5]. After a cascade of reactions, this leads on the one hand to the inhibition of tumor growth (action on the secretion of hormones) and blocking proliferation via the activation of different tyrosine phosphatases (anti-proliferative and pro-apoptotic action), but also to the inhibition of the secretion of growth factors such as growth hormone or IGF-1 having a major role in the inhibition of tumor growth (anti-angiogenic) (Figure 2) [6,7].

Over the past 20 years, our understanding of the phenomena due to the activation of SSTRs has increased thanks to numerous translational and clinical studies, leading to the development of new therapeutic options [3]. The use of SST analogs has demonstrated real effectiveness in the treatment of various pathologies: acromegaly (production of an excess of growth hormone), pancreatitis, complications linked to diabetes and obesity (e.g., retinopathy or nephropathy), action on inflammation and pain in some cases [5,9]. However, SSTRs and SST analogs are mainly known for their presence and role in the detection and treatment of some solid tumors. Tumor cells and peritumoral vessels express receptor subtypes whose density depends on the type of tumors (Table 1) [10,11,12,13]. For those overexpressing SSTRs, such as pituitary adenomas, gastroentero-pancreatic neuroendocrine tumors (GEP-NET), or other cancers (e.g., lymphomas, small cell lung cancers, etc.), targeting with SST analogs becomes possible [14]. Many therapeutic protocols based on these analogs (classic octreotide or with a longer release time (octreotide LAR), Lanreotide, Vapreotide, Pasireotide, etc.) have been the subject of phase II and III clinical trials. The majority of results were generally disappointing and did not provide clear evidence of a significant antitumor effect on solid tumors, probably due to the existence of other pathways of tumor progression [15,16].

For example, regarding liver tumors, such as hepatocellular carcinoma (HCC), in vitro studies clearly demonstrated (i) the lack of SSTRs expression in healthy liver cells; (ii) overexpression in tumors and metastases of HCC, even though their density is less than in neuroendocrine tumors [21,22]. On the other hand, the results show a heterogeneous expression and strong inter-individual differences. In fact, according to studies, HCCs express high levels of SSTR2 [21,23,24] or SSTR5 [13,19], or even SSTR1 [22] or SSTR3 [25]. In general, around 40% of HCCs studied express somatostatin receptors. These differences could be due to the different methodologies used during the measurements, by studying different stages of the disease or even by heterogeneous behaviors of HCC. Further studies have also found a correlation between the density of SSTRs expression, disease aggressiveness [26], and the rate of tumor recurrence after treatment with octreotide LAR [27]. In a study by Nguyen-Khac et al. [23], 41.2% of extrahepatic metastases express SSTR2. Preclinical tests on HCC cell lines have shown an antiproliferative effect of SST analogs [25,28]. In addition, a real decrease in invasion and cell migration of HCC cells after stimulation of SSTR1 by a specific agonist has also been demonstrated [22]. This action has also been confirmed in vivo [29], with the demonstration of a similar effect on metastatic dissemination [23,30]. These initial results paved the way for clinical trials on patients with HCC, but their conclusions are quite contradictory, [31] showing rather positive effects in the advanced stages [32,33] and others quite negative [34,35]. These outcome discrepancies could come from heterogeneity in the choice of patients, but available data are still insufficient to truly conclude on the effectiveness of analogs of SST alone in the control of HCC tumors [6,31,36]. Cholangiocarcinoma, the other main primary liver tumor, might also be a potential target [18,37].

On the other hand, in certain cases, and in particular for neuroendocrine tumors (a category of tumors where SSTRs are the most expressed), a benefit has been proven via two Phase III studies, which have greatly contributed to the fact that SST analogs are now used in clinical routine [38,39].

## 2. Somatostatin Analogs

**Figure 3:**
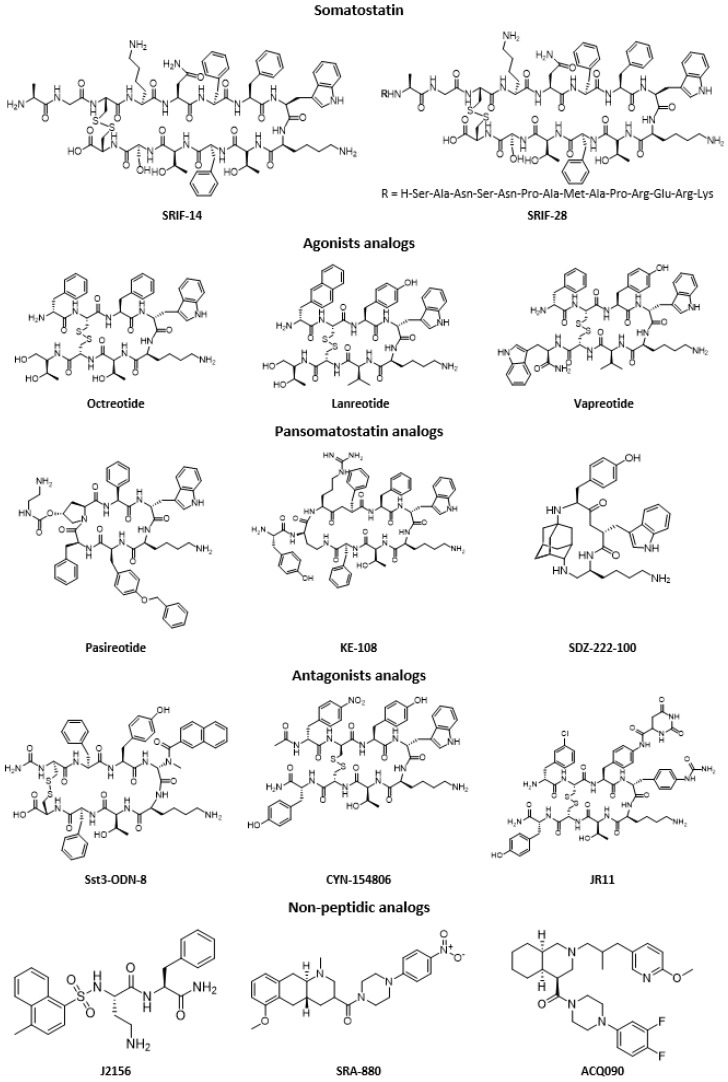
Chemical structures of SRIF-14, SRIF-28, and selected examples of somatostatin analogs.

Somatostatin has a short half-life in the body (between one and three minutes), because it is rapidly degraded by peptidases found in plasma and tissues [40]. Therefore, the amount present in the bloodstream is extremely low (between 14 and 32.5 pg/mL). This very short half-life has been considered a limiting factor for possible clinical applications, thus many analogs with better metabolic properties (longer half-life between 1.5 h and 12 h) have been rapidly developed [2,5,9]. These are most often hexapeptide or octapeptide molecules which incorporate the biologically active core of native somatostatin (see some examples in Figure 3). Indeed, studies on the structure–activity correlation have shown that the Phe^7^, Trp^8^, Lys^9^, and Thr^10^ sequence in the form of a β-sheet is necessary for biological activity. The residues Trp^8^ and Lys^9^ are essential for this activity, whereas Phe^7^ and Thr^10^ may undergo some substitutions. Among somatostatin analogs, there are two main categories: the agonists (substances capable of activating somatostatin receptors) and the antagonists (molecules that interact with somatostatin receptors and block or reduce the physiological effect of an agonist). It is also important to note that somatostatin analogs have different affinities for the different receptor subtypes [2].

The first agonist peptide analog to be approved by the FDA was octreotide (SMS 201-995), marketed under the name Sandostatin^®^. From a structural point of view, it has a d-Trp and a d-Phe, to stabilize the β-sheet and a disulfide bridge closer to the active core, for a better metabolic stability. Its pharmacodynamics is highly similar to native SST, which has made it widely used in clinical trials for the treatment of GEP (gastro-entero-pancreatic) tumors [41,42]. Next, Lanreotide (BIM 23014, tradename Somatuline^®^), whose structure is similar to that of octreotide (Phe and Thr having been replaced by Tyr and Val respectively), showed comparable characteristics and is also widely used in the treatment of neuroendocrine tumors [43]. In 2005, another analog, Vapreotide (RC160), was marketed under the name Sanvar^®^, with properties close to those of the two previous analogs, and is also used for the treatment of esophageal varices. More recently, Pasireotide (SOM-230 or Signifor^®^) was one of the first analogs to show a strong affinity for most of the somatostatin receptor subtypes (pansomatostatin analog). Marketed by Novartis, it is used for the treatment of Cushing’s disease [44]. Many other analogs have been developed, from “ultra-short” peptides, such as SDZ 222-100 (an adamantine cyclopeptide), to longer ones, such as KE-108 or CH-275 [5]. Regarding antagonist peptide analogs, the wide variety of compounds that the octapeptide model can offer has allowed the discovery of several structures that can block this kind of receptors. The first antagonist that has been described in the literature is CYN-154806, followed by PRL-2970, sst3-ODN-8 or even non-cyclic models such as BIM-23056 and BIM-23627. New non-peptide compounds have also emerged [45]. These agonists and antagonists (selective or not) constitute a very promising field in the chemistry of somatostatin analogs, in particular because of their pharmacological, pharmacokinetic, and physicochemical properties. This type of compound may have a stronger affinity and/or selectivity for certain subtypes of somatostatin receptors than the majority of peptide analogs. They can thus provide additional information on the exact role of each of these subtypes [5,9].

## 3. Targeting of Somatostatin Receptors with Radiopharmaceuticals

**Figure 4:**
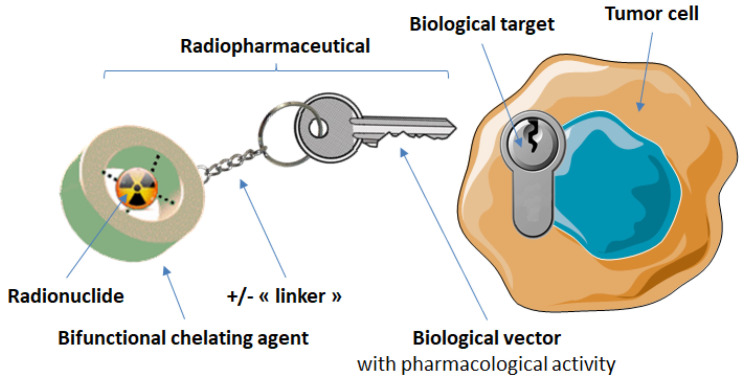
Schematic design of a radiometallated bioconjugate.

In the field of medicine, much research is focused on finding methods to achieve earlier detection of pathologies to allow treatment at early stages of the disease, to increase the chances of total recovery. For this purpose, nuclear medicine, through the use of radiopharmaceuticals, is a very powerful tool. Its application can have two different aims: imaging, with the visualization of a radioactive element’s distribution in the body, or therapy, with specific irradiation of abnormal cells, thereby reducing damages to nearby healthy tissue. Having a broad range of potential biological targets and desirable pharmacokinetic characteristics—such as high uptake in target tissue and fast blood and non-target tissue clearance—peptides can also be easily chemically modified for incorporation into a radiopharmaceutical, making them a very potent targeting vector for nuclear medicine. Research in that domain has thus gained widespread interest [46,47,48,49]. These compounds can be directly labeled with a radionuclide, such as a halogen radioisotope, but they are generally based on a triple structure involving: (i) a radiometal, the radiation of which allows either the localization (γ and β^+^ emitters) or the destruction (β^−^, α or Auger electron emitters) of the targeted cells; (ii) a bifunctional chelating agent (BFCA), the dual role of which is not only to bind the radiometal in a very stable manner to minimize its dissociation in vivo, but also to allow its conjugation with targeting moiety (or vector) via a functionalized arm; (iii) a targeting moiety (the peptide analog), which aims to convey this set in a specific way to a well-defined target. To limit the influence of the chelating moiety, a linker (or spacer) is usually inserted between the BFCA and the biomolecule (Figure 4).

The choice of the radiometal is crucial, since it deeply influences the design of the chelating structure [50,51,52,53]. Several criteria govern the choice of radionuclide: (i) the nature of the radiation emitted, depending on the intended application (diagnosis or therapy); (ii) the half-life, which must be long enough to allow effective fixation of the radiotracer on the target cells, but relatively short to avoid irradiation of the organism (neighboring healthy tissues) and more specifically non-targeted organs; (iii) the isotope decay profile. By emitting its radiation, the nuclide disintegrates into a daughter nuclide, which must be non-radioactive to avoid any additional harmfulness to the organism; (iv) the means of production. Most of the radioelements used in nuclear medicine are artificial. They can be produced in three different ways: from a nuclear reactor, a cyclotron or via a generator. Generator production remains the most convenient way for clinical application, as it can provide in-house radionuclides when a cyclotron is not available nearby, but cyclotron production still remains the cheapest and most used. As an example, Table 2 shows some of the characteristics of radioactive nuclides among the most used today for the radiolabeling of peptides.

From a structural point of view, each radiometal has its own properties such as polarizability, degree of oxidation, or coordination number. These features have a direct impact on the choice of the bifunctional chelating agent, in particular in terms of denticity and nature of the donor atoms (most often *O*-, *N*-, or *S*-donors) [54,55]. The BFCA makes it possible to link the biomolecule and the radiometal; its choice is a crucial step in the construction of a radiopharmaceutical. As indicated above, this structure plays a double role: the first is to complex the radioelement in a very stable manner. Several criteria can be evaluated to truly attest to the stability of the complex formed. First of all, the formed radiocomplex must be thermodynamically stable, i.e., the metal-ligand affinity must be as strong as possible. Then it must be kinetically inert. Many metalation reactions take place in the body and the complex formed must be stable enough to avoid any in vivo degradation (e.g., demetallation or transchelation). In addition, radiolabeling conditions with low concentrations are required, ideally with efficient complexation kinetics (high labeling yield) and fast and mild reaction conditions. Beside chemistry considerations, the radiotracer must have: (i) a strong affinity for the target receptor; (ii) a high accumulation for the target and low for the non-target organs; (iii) relatively rapid clearance in the organism; (iv) preferably a mainly renal route of excretion.

**Figure 5:**
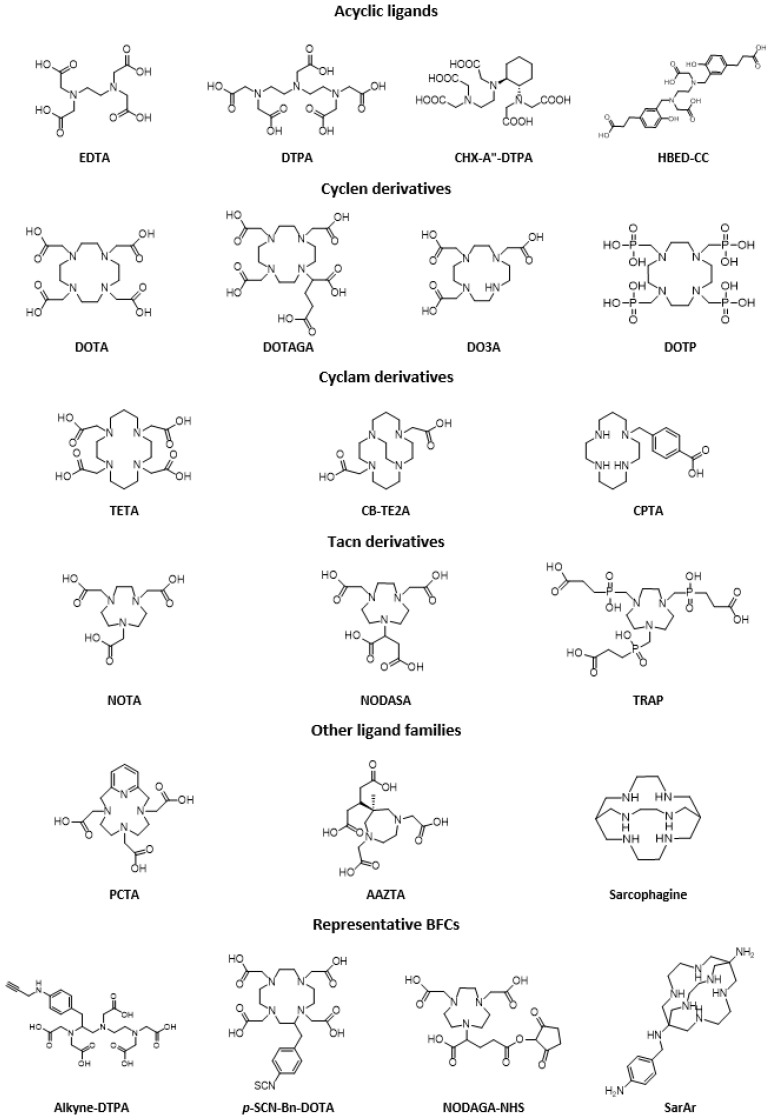
Representative (but not exhaustive) examples of acyclic and macrocyclic polyamino and polyaminocarboxylic chelator families and their derivatives.

Chelating ligands used for the design of radiotracers are usually classified into two categories: macrocyclic and acyclic compounds (Figure 5). Generally, acyclic ligands are less kinetically inert than macrocycles, although some may have shown very good characteristics. On the other hand, these ligands generally have faster metal-chelate binding kinetics compared to macrocyclic analogs, which represents a huge advantage for working with isotopes that have a short lifespan. Despite the coordination properties specific to each metal, some chelating agents—such as polyaminopolycarboxylic acids—are considered to be ‘universal’ because they can complex different radiometals.

Among acyclic ligands, the first BFCAs developed were EDTA (ethylenediaminetetraacetic acid) and DTPA (diethylenetriaminepentaacetic acid). They have been widely used in the chemistry of radiopharmaceuticals, in particular with radioelements such as ^111^In, ^90^Y or ^177^Lu, and even ^99m^Tc [54]. Later on, DTPA derivatives such as CHX-A′′-DTPA with a cyclohexyl moiety bringing more rigidity to the DTPA backbone (allowing a pre-organization of the system) showed better kinetic inertia [56]. Regarding cyclic compounds, cyclen derivatives such as DOTA (1,4,7,10-tetraazacyclododecane-1,4,7,10-tetraacetic acid) and triaza analogs—NOTA (1,4,7-triazacyclononane-1,4,7-triacetic acid)—are among the most studied ligands. NOTA has the smaller chelating cavity of the two, and is generally used for Ga (III) or Cu (II) because it has a particular attraction for these metals, which results in mild radiolabeling conditions and good in vivo stability of the complexes formed. DOTA (which is considered as the gold standard chelator) and its derivatives play an important role in clinical applications because they form very stable complexes with a wide range of trivalent radiometals such as Ga (III), Y (III), In (III), Lu (III), or even divalent such as Cu (II) [57,58]. For DOTA or NOTA, the introduction of a functionalized arm offers the possibility of coupling a biomolecule (NODASA/NODAGA and DOTASA/DOTAGA). Similarly, TETA (1,4,8,11-tetraazacyclotetradecane-1,4,8,11-tetraacetic acid), has mainly been studied with Cu (II) and have shown a stability similar to DOTA [59].

Whether on the side of macrocyclic ligands, derivatives or variations of DOTA (e.g., p-SCN-Bn-DOTA, DOTAGA, CB-DO2A, TCMC…), NOTA (e.g., p-SCN-Bn-NOTA, NETA…), or TETA (e.g., CB-TE2A, p-NH2-Bn-TE3A…), or on the side of acyclic ligands, derivatives or variations of DTPA (e.g., CHX-A′′-DTPA…), a large number of ligands have been developed so far. A wide choice of ligands is available for the design of new agents, and numerous journals have described and carefully classified all the structures that can be used in the design of a radiopharmaceutical, whatever the intended application [46,51,53,54,57,60].

BFCA’s second role is to allow the conjugation of the complex with a biomolecule. The nature of this link is very important, because it is essential for it to be stable, and above all, for it to not interfere in any way with the binding to the receiver. The slightest structural modification of the ligand and/or of the biomolecule can have a very marked effect on the affinity to the targeted receptors. To minimize this impact as much as possible, that sometimes a ‘spacer’ or ‘linker’ can be used between these two entities. Biomolecules are often functionalized through a primary amine, which provides an ideal conjugation site for a coupling reaction, most often with peptide or thiourea type links. Other links based on thioether, triazole, oxime, or more recently via a copper-free click-chemistry with tetrazine/cyclooctyne may prove to be interesting, in particular, because they have very good stability in vivo [51,54,61].

Many somatostatin analogs have already been labeled with various radioelements, whether for imaging, with probes used today in clinical applications, or for therapy, with many compounds in clinical studies [17,61,62,63]. These analogs were obtained from modifications in the sequence of amino acids that make up the peptide. For example, replacing Phe^3^ in octreotide (OC) with Tyr^3^ (TOC) improves the affinity for SSTRs (in particular SSTR2) and introduction of a Thr (TATE) instead of Thr(ol) (TOC) further improves this. By following this procedure, many analogs have been developed and studied, often with the same chelating cavity to be able to compare their properties (Table 3) [64,65].

### 3.1. Radiolabeled Somatostatin Analogs for Imaging

**Figure 6:**
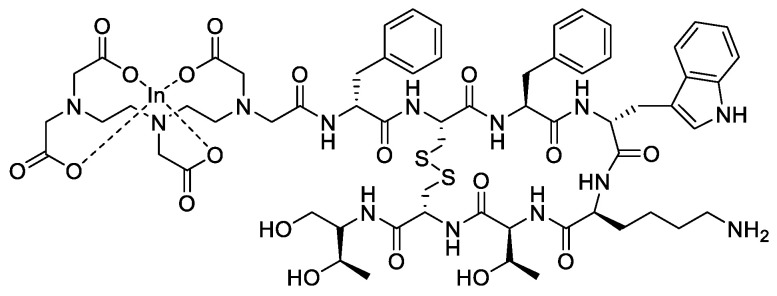
Structure of [^111^In]-pentetreotide (Octreoscan^®^).

The very first proof of concept for the visualization of tumors expressing SSTRs was carried out with [^123^I-Tyr^3^]-octreotide, obtained from an iodination reaction (electrophilic substitution) of tyrosine [66,67]. This compound demonstrated biological activity and an affinity for receptors similar to those of native SST [68]. Despite the obvious interest of this probe, several factors such as the difficult radiolabeling procedure, the significant cost, and particularly, the clearance via the liver and the hepatobiliary system (which makes it difficult to interpret the obtained images) were the main drawbacks of its application [67]. To overcome all of these disadvantages, iodine-123 has been replaced with indium-111, which, through the chelating agent DTPA, has been coupled to octreotide (Figure 6) [69]. In vivo studies of [^111^In-DTPA^0^]-octreotide ([^111^In]-pentetreotide) have shown that it is possible to visualize tumors expressing SSTRs and their metastases, even 24 h after injection. In comparison with the compounds coupled to antibodies, this reveals a relatively rapid clearance via the kidneys, which represents a huge advantage compared to [^123^I-Tyr^3^]-octreotide [70,71]. This compound was the first radiopharmaceutical targeting SSTRs to be approved by the FDA (Octreoscan^®^ marketed in 1994). It has been widely used, and has long been considered a ‘gold standard’ for the visualization of neuroendocrine tumors. It still has a few limits: in fact, it requires a high tumor/noise intensity ratio, shows low spatial resolution, has a moderate affinity for receptors and finally, and possesses a high γ energy which results in a high dose of radioactivity received by the patient. For all these reasons, research in the field of radiopharmaceuticals has focused on other radioelements such as technetium-99m for SPECT and gallium-68 for PET. In addition to having excellent physical properties, these two elements are available from a commercial clinical-grade generator, an important advantage for clinical applications.

#### 3.1.1. Gallium-68 and Indium-111

DOTATOC analog was the first to be radiolabeled with indium-111, and its comparative study with Octreoscan^®^ showed similar diagnostic accuracy, but with better biodistribution and clearance [72]. Although DOTATATE alone showed better affinity for SSTRs, the two analogs [^111^In]-DOTATOC and [^111^In]-DOTATATE showed relatively similar pharmacokinetic properties [73]. SSTR2 receptors—and to a lesser extent, SSTR5—are most often overexpressed in tumors. Consequently, the majority of the radiotracers described have a strong affinity for these two SSTRs subtypes. Systems such as DOTANOC were designed to develop a probe capable of targeting all subtypes. Compared to DOTATOC and DOTATATE, it has a similar affinity for SSTR2 and SSTR5 subtypes, but a much higher affinity towards SSTR3. Their high internalization rate results in interesting biodistribution data, with a greater accumulation of the probe in the tumor and in the organs or tissues expressing SSTRs (e.g., pancreas and adrenal glands), ending with excretion mainly by kidneys [74].

**Figure 7:**
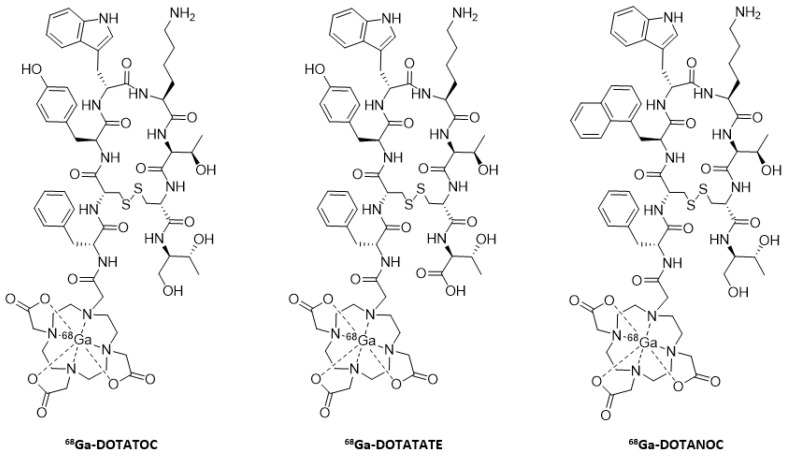
Structures of the three main systems radiolabeled with gallium-68.

These three systems, similarly labeled with gallium-68 (Figure 7), have proven to be very good radiotracers, and are currently routinely used in clinical applications [75]. These three radiopharmaceuticals have slightly different pharmacokinetic properties, but their diversity is mainly due to the variation in affinity for certain subtypes. This feature is even more marked depending on the radioelement chosen (^68^Ga or ^111^In). This can be explained by the differences in the geometry of the complexes. [^68^Ga]-DOTATOC is very affine for SSTR2 and more moderate for SSTR5, [^68^Ga]-DOTATATE is specific to SSTR2 and finally, [^68^Ga]-DOTANOC binds with great affinity to SSTR2, SSTR3, and SSTR5 [76,77,78].

A study with DOTANOC aimed at determining the impact of the introduction of a spacer on the pharmacokinetic properties of the formed radiotracer. The aim was to insert polyethyleneglycol (PEG) moieties or sugars between the chelating cavity (DOTA) and the biomolecule (NOC), which resulted in the modification of the lipophilicity or the charge of the final compound. As a result, the hydrophilicity of the system seems to be involved only in the affinity phenomenon towards the receptor, and the overall charge of the compound influences the excretion profile [79].

DOTA is not the only macrocycle to have been coupled to somatostatin analogs. Knowing the attraction of Ga (III) for NOTA, the latter has been the subject of comparative studies. Conjugated with octreotide (NODAGATOC), the compound showed a strong affinity for SSTR2 (similar to that of DOTATOC). Once marked with ^111^In, affinity was even stronger for SSTR2, with even a gain on SSTR3 and SSTR5 (compared to ^68^Ga-NODAGATOC), which confirms the influence that the geometry of the complex can have on affinity. In terms of stability, as expected, that of [^68^Ga]-NODAGATOC was higher than that of [^111^In]-NODAGATOC. The biodistribution of [^68^Ga]-NODAGATOC was similar to that of [^68^Ga]-DOTATOC, but showed a better accumulation in the tumor than [^111^In]-DOTATOC. This is probably due to the strong agonist character, and the high rate of internalization of the NODAGATOC derivative [80].

A large variety of derivatives have also been investigated, such as DOTALAN, DOTABOC, DOTAGA [81], DOTANOCATE or DOTABOCATE (all derivatives of DOTANOC) [82,83], or THP-TATE (comparison of the overall behavior of the tris chelating system (hydroxypyridinone) with DOTATATE) [84]. New generation analogs with broader affinity profiles or pan-somatostatin analogs have been developed. For instance, AM3 (DOTA-Tyr-cyclo(DAB-Arg-cyclo(Cys-Phe-d-Trp-Lys-Thr-Cys))), a bicyclic somatostatin analog demonstrated affinity to SSTR2, 3, and 5, when labeled with ^68^Ga. It showed a fast background clearance coupled with a high tumor/non-tumor ratio. [85] KE108 was coupled with DOTA and labeled with ^111^In and ^68^Ga, giving [^111^In/^68^Ga]-KE88 (DOTA-d-Dab-Arg-Phe-Phe-d-Trp-Lys-Thr-Phe), which bound to all five SSTRs with high affinity. [86] However, in an in vitro study, it had a low SSTR2 uptake, but was very effective for SSTR3-expressing tumors. More recently, a Pasireotide derivative, DOTA-PA1 (DOTA-cyclo-[HyPro-Phe-d-Trp-Lys-Tyr(Bzl)-Phe]) was labeled with ^68^Ga and was investigated in three human lung cancer models, where it demonstrated superiority compared to [^68^Ga]-DOTATATE [87]. In parallel, the group from Demokritos Institute, in Athens, developed pansomatostatin radiopeptides based on native somatostatin (SRIF-14 and SRIF-28). Both were derivatized with DOTA chelator and labeled with ^111^In. Subsequent radiotracers exhibited high affinity and internalization profiles. SRIF-14 derivatives unfortunately demonstrated low in vivo stability. [^111^In]-DOTA-LTT-SS28, on the contrary, demonstrated a much higher stability and showed more promise [88,89].

#### 3.1.2. Technetium-99m

**Figure 8:**
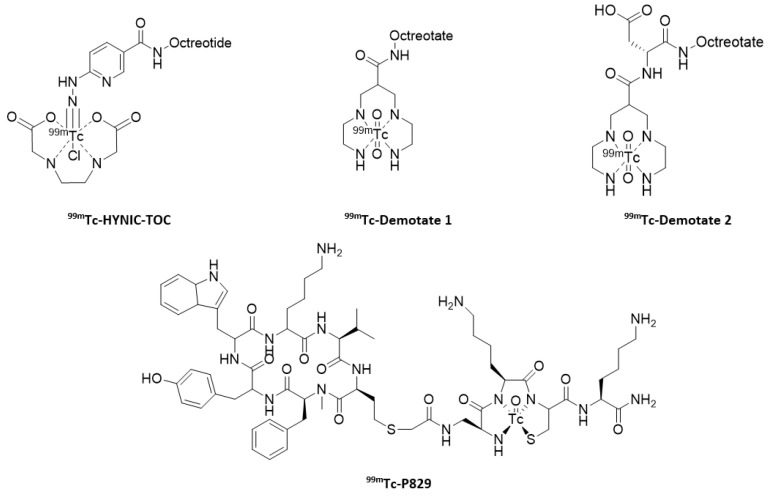
[^99m^Tc]-labeled somatostatin analogs.

A wide range of chelating agents have been used to prepare somatostatin analogs labeled with technetium-99m: peptide moieties [90,91], propyleneaminooxime [92], tetraamines [93,94] or a cyclopentadienyl group [95]. Macrocyclic ligands have also been investigated [96]. Three systems stand out for the radiolabeling of somatostatin analogs: HYNIC-TOC and Demotate scaffolds, and P829 (Figure 8).

Initially, the HYNIC core (hydrazinonicotinamide) was designed for the radiolabeling of antibodies and proteins with technetium-99m [97], then this was transposed to peptides and more specifically to octreotide. This ligand can complex the metal in a monodentate or bidentate way, therefore, it is necessary to use one or more co-ligands to complete the coordination of the [^99m^Tc]-HYNIC core. Among the most commonly used co-ligands are tricin, nicotinic acid, or EDDA (ethylenediaminodiacetic acid). Each co-ligand has its own influences on the properties of the complex obtained (e.g., lipophilicity and biodistribution) [98]. The first studies were carried out using tricin as a co-ligand ([^99m^Tc]-HYNIC-TOC), but quickly EDDA demonstrated a very favorable influence on the pharmacokinetics of the radiotracers [99]. Compared to Octreoscan^®^, [^99m^Tc]-EDDA/HYNIC-TOC showed better accumulation in the tumor and a weaker accumulation in the kidneys. The improved spatial resolution, the reduction in the radiation dose received by the patient and the better availability of ^99m^Tc made it a possible alternative to Octreoscan^®^ [99,100]. Finally, its conjugation with the octreotate analog ([^99m^Tc]-EDDA/HYNIC-TATE) has shown significantly similar behavior to its octreotide counterpart [101]. [^99m^Tc]-EDDA/HYNIC-TOC (Tektrotyd^®^) was granted marketing authorization in Europe in adult patients with gastro-enteropancreatic neuroendocrine tumors (GEP-NET) for localizing primary tumors and their metastases.

The second radiotracer, based on the tetraamine motif 6-R-1,4,8,11-tetraazaundecane, is available in a series with [^99m^Tc]-Demotate 1 ([^99m^Tc-N_4_^0^, Tyr^3^]-octreotate) and ^99m^Tc-Demotate 2 ([^99m^Tc-N_4_^0–1^, Asp^0^, Tyr^3^]-octreotate). The first version of this probe demonstrated excellent pharmacokinetic properties, including faster accumulation in the tumor compared to Octreoscan^®^ [102]. The objective of the second version was to improve the qualities of [^99m^Tc]-Demotate 1, by modifying the overall charge of the complex and adding an Asp residue. In the end, [^99m^Tc]-Demotate 2 showed overall behavior similar to [^111^In]-DOTATATE, even if the latter has a faster clearance and a better retention time in the tumor [103]. The last of the main analogs based on technetium-99m is [^99m^Tc]-P829 (^99m^Tc-Depreotide), marketed in 2000 by the company CISBio International under the name of NeoSpect^®^, but recently withdrawn from the market. The P829 peptide (directly radiolabeled with ^99m^Tc) showed results similar to the other SST analogs [104]. Its use for the detection of neuroendocrine tumors appeared to be less precise than with Octreoscan^®^ [105]. On the other hand, its affinity for SSTR3, subtype which may be the origin of cross-competition from other types of receptors (notably VIP receptors), gave it the ability to bind to a larger number of primary tumors [104]. In particular, it was used clinically for the diagnosis of malignant lung tumors [106,107,108], for which it got its market authorization [109], and also demonstrated some interest in breast cancer, but it was never confirmed in a larger series of patients [110].

The question that now remains to be answered is that of the clinical interest of a SPECT tracer among the wide choice of PET SSTRs imaging agents [111,112].

#### 3.1.3. Copper-64

Due to the short half-life of ^68^Ga (T_1/2_ = 67.7 min.) each center willing to perform ^68^Ga PET imaging must purchase a currently expensive ^68^Ge/^68^Ga generator and a specifically shielded hot-cell. For this reason and despite the FDA and EMA market authorizations for [^68^Ga]-DOTATATE and [^68^Ga]-DOTATOC and the better diagnostic performances for these two radiopharmaceuticals products, the use of ^68^Ga appears to be under the dependence of an economic choice for many hospitals and only a few large centers are making the financial investment to perform ^68^Ga-radiolabeling. In this context, the use of a PET-emitter with a longer half-life such as copper-64 (T_1/2_ = 12.7 h) appears to be an interesting alternative to remove the financial hindrance of gallium-68 [113]. This physical parameter allows for a centralized radiolabeling site with a large multicentric supply of ready-to-use ^64^Cu-radiolabeled compounds. The chemistry of copper is also well known, which is a real asset in the design of new radiotracers. Many systems already presented before, such as DOTATOC/TATE or NODAGATOC/TATE, or others more copper-specific BFCAs, such as TETA (1,4,8,11-tetraazacyclotetradecane-*N*,*N*′,*N*″,*N*‴-tetraacetic acid) [114], and its more stable derivatives such as cross-bridge CB-TE2A (4,11-bis(carboxymethyl)-1,4,8,11-tetraazabicyclo [6.6.2]hexadecane) [115], and CPTA (4-[(1,4,8,11-tetraazacyclotetradec-1-yl)methyl]benzoic acid]) [116] or sarcophagine derivatives [117] have been studied. A review on the development of copper radiolabeled somatostatin analogs was recently published by Marciniak et al. [118].

To validate the clinical interest of [^64^Cu]-somatostatin analogs, various clinical studies have been conducted around the world. Among the different somatostatin analogs, [^64^Cu]-DOTATATE was one of the first used. In 2015, [^64^Cu]-DOTATATE was compared head-to-head to [^111^In]-DTPA-octreotide in 112 patients and showed that the PET ^64^Cu-compound was far superior to SPECT ^111^In compound performances [119]. In 2017, [^64^Cu]-DOTATATE was challenged to [^68^Ga]-DOTATOC according to an identical PET/CT imaging modality [120]. The results of this study, where 59 patients were injected with [^68^Ga]-DOTATOC followed by an injection of [^64^Cu]-DOTATATE one week later, concluded that the two radiopharmaceuticals had the same sensitivity. Nevertheless, in this cohort of neuroendocrine tumors, [^64^Cu]-DOTATATE had a substantially better lesion detection rate. The patient follow-up revealed that these additional lesions detected by [^64^Cu]-DOTATATE were true positives. To evaluate the benefits of this better detection of lesions with [^64^Cu]-DOTATATE than with [^68^Ga]-DOTATOC, the correlation between PET image [^64^Cu]-DOTATATE uptake (expressed in maximal standardized uptake value - SUV_max_) and overall (OS)/progression free survival (PFS) was studied during 24 months after [^64^Cu]-DOTATATE PET/CT acquisition. The conclusion of this study claimed a good correlation/prognostic between SUV_max_ and PFS but not with OS [121]. The major drawback of these preliminary human studies consist of the affinities differences for the five SSTRs subtypes between DOTATOC and DOTATATE compounds. To circumvent these discrepancies, an in vitro study in a mouse model was conducted and compared [^64^Cu]-DOTATATE to [^68^Ga]-DOTATATE. The results showed a similar pharmacokinetic and absolute uptake between both compounds 1 h post-injection [122]. In Europe, where the PET radiopharmaceutical approved is [^68^Ga]-DOTATOC, it could be interesting to perform some PET imaging with [^64^Cu]-DOTATOC to compare the performance of the two tracers. A first-in-human retrospective study was recently conducted and seems to present same results than [^64^Cu]-DOTATATE with high detection rate of suspected lesion associated to a high target-to-background contrast [123]. A recent first-in-human study also demonstrated potential interest for [^64^Cu]-SARTATE analog [124].

In conclusion, despite a higher dosimetric impact for copper-64 (only 17.6% of radioactive decay lead to positron emission), copper-64 somatostatin analogs appear to be an advantageous alternative to gallium-68 radiopharmaceuticals. Compared to ^68^Ga, in addition to economic advantages, ^64^Cu has a lower positron range which leads to a better PET intrinsic resolution and a higher half-life which allows for a more flexible scanning window. The better patient care management and outcomes remain to be proven and the work is in progress to establish these points [121,125]. In parallel, at present, a radiopharmaceutical industrial company submitted a market authorization from FDA for [^64^Cu]-DOTATATE and thus confirms the interest of copper-64 in SSTRs imaging.

#### 3.1.4. Other Radiometals

Other radionuclides have also been investigated for SSTRs imaging. Cobalt-55 seems to be a possible alternative to gallium-68 and copper-64 compounds, with similar behavior and lifespan (17.5 h vs. 12.7 h) to the latter, but with a higher positron yield (75.9% vs. 17.6%). Preliminary complexation tests of DOTATOC with the isotope ^57^Co as a surrogate for ^55^Co showed a higher affinity for SSTR2 than [^68^Ga]-DOTATOC, implying a rate of internalization among the highest of all derivatives of SST and thus, a strong accumulation in targeted tissues. Despite similar structures, the analogs of cobalt and gallium have different biological behaviors. This confirms the fact that the physical characteristics of radioactive elements influence the affinity, biodistribution, and pharmacokinetics of radiolabeled peptides [126]. The properties of cobalt-based compounds have been further investigated with the comprehensive evaluation of other octreotide analogs such as DOTANOC and DOTATATE [127]. Furthermore, [^55^Co]-DOTATATE compared favorably with [^68^Ga]-DOTATATE and [^64^Cu]-DOTATATE in an animal model [122]. Associated with the Auger-emitting ^58m^Co, it could represent a potentially interesting theranostic pair [128].

Scandium and terbium are two metals that recently emerged as possibly useful for theranostic applications, as both possess imaging and therapeutic radionuclides [129]. DOTATOC was radiolabeled with scandium-44 (T_1/2_ = 3.97 h, E_β+_ = 632 keV) [130] and terbium-152 (T_1/2_ = 17.5 h, E_β+_ = 1140 keV) [131] and rapidly injected in patients in proof-of-concept studies [132,133]. No adverse effects were observed during follow-up periods and images proved suitable for diagnosis. With DOTATATE, it seems the affinity to SSTR2 receptors is lower with scandium than with gallium, thus limiting its interest [134]. In a study comparing the labeling and stability of DOTANOC and NODAGANOC with ^44^Sc and ^68^Ga, it was observed that [^44^Sc]-NODAGANOC labeling was more challenging and less stable than [^44^Sc]-DOTANOC [135]. The opposite was observed with ^68^Ga. Recently, a new chelator was proposed, AAZTA (1,4-bis (carboxymethyl)-6-[bis (carboxymethyl)]amino-6-methylperhydro-1,4-diazepine), which enables fast and easy labeling at room temperature. AAZTA-TOC labeled with ^44^Sc demonstrated high in vitro stability [136]. Affinity tests are now necessary to assess its potential utility. DOTATATE has also been labeled with ^155^Tb (T_1/2_ = 5.32 days, E_γ_ = 87 keV (32%), 105 keV (25%)) for SPECT imaging [137]. Though a potentially promising radionuclide for theranostic applications, availability of ^155^Tb is currently the main limitation for further development.

At the turn of the millennium, yttrium-86 (T_1/2_ = 14.74 h, 32% β^+^) was thought to be a potential radionuclide of interest, particularly for pretherapeutic dosimetry of ^90^Y-radiotracers, and notably ^90^Y-labeled somatostatin analogs [138]. Thus, several octreotide analogs were developed [139,140]. [^86^Y]-DOTATOC even reached the clinics [139,141]; however, ^86^Y properties are less than optimal, and availability is limited, so interest soon faded out.

#### 3.1.5. Fluorine-18

**Figure 9:**
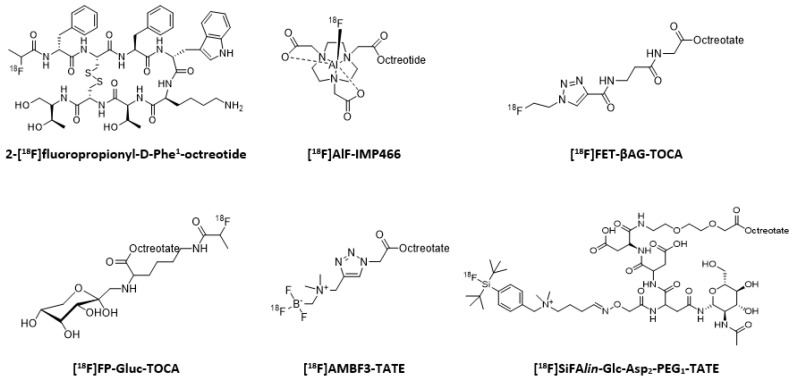
[^18^F]-labeled somatostatin analogs.

Radiometals’ production is currently still limited, even for the most advanced ones [142,143,144]. Fluorine-18, on the contrary, can be mass-produced and distributed daily, thanks to a worldwide network of cyclotrons. Because of this availability, and favorable decay characteristics (T_1/2_ = 110 min, 97% β^+^), it thus should be noted that some radiotracers based on fluorine-18 have been described (Figure 9) [145]. The first generations such as 2-[^18^F]fluoropropionyl-d-Phe^1^-octreotide [146] or 4-[^18^F]fluorobenzoyl-d-Phe^1^-octreotide [147] generally showed unfavorable biokinetic properties (low accumulation and low retention in the tumor). The probes developed subsequently contained hydrophilic or charged moieties to reduce the lipophilicity of the radiotracer. In particular, several carbohydrate derivatives of octreotide/octreotate have been developed [148,149]. A disadvantage of fluorine-labeling compared to radiometal labeling is the use of generally long and tedious multi-step procedures. To circumvent this, innovative strategies, enabling fast and purification-less labeling, have been developed, such as the formation of ^18^F-boron or ^18^F-silicon bonds, or the use of click-chemistry [150,151,152]. Another elegant method to label somatostatin analogs is the use of [^18^F]-aluminum fluoride with radiotracers previously developed for radiometals, such as NOTATOC [153]. These new generation analogs demonstrated general properties (affinity for the targeted receptors, metabolic stability, biodistribution and clearance) which are much more interesting, and some of them have been investigated in patients, where they gave results comparable to [^68^Ga]-DOTATOC [154,155]. In addition, [^18^F]F-FET-βAG-TOCA and [^18^F]-IMP466 ([Al^18^F]-NOTATOC) are currently being evaluated in phase I clinical trials (EudraCT number 2013-003152-20 and NCT03511768, respectively). Recently published results with [^18^F]-IMP466 demonstrated it was safe and well-tolerated, with a physiologic uptake pattern similar to [^68^Ga]-DOTATATE [156]. Besides cost and availability, another advantage of fluorine-18 is its shorter positron range compared with gallium-68, leading to an improved spatial resolution, and thus, better quantification of uptake [157].

### 3.2. Radiolabeled Somatostatin Analogs for Therapy

Concerning radionuclide therapy and more particularly peptide receptor radionuclide therapy (PRRT), radioactivity is used to destroy the targeted cells. Radiopharmaceuticals used in therapy are designed in the same way as those used in imaging, only the nature of the radioelement being modified. Contrary to imaging, which uses radioelements having very penetrating but little ionizing radiations, PRRT privileges the use of radionuclides that have little penetrating and more energetic and thus more ionizing radiations. Brought directly to the cancer cell, the radiation emitted by the radioactive decay causes irreversible ionization of the cell’s DNA, which induces its apoptosis. The main isotopes used today are iodine-131, yttrium-90, lutetium-177 and, to a lesser extent, rhenium-188 [158]. As mentioned earlier, the purpose of the DOTA-SSA design was to work with a chelating cavity capable of complexing radioelements for imaging or therapy. Consequently, most of the platforms discussed above have been transposed for therapeutic application via the use of β^−^ emitters [64,74,81,82].

#### 3.2.1. Yttrium-90 and Lutetium-177

Yttrium-90, a pure high energy β^−^ emitter (T_1/2_ = 64 h, E_βmax_ = 2.28 MeV), and lutetium-177, a medium energy β^−^ emitter (T_1/2_ = 6.7 d, E_βmax_ = 0.5 MeV) with a γ component (208 keV), are currently the most used in PRRT. Each of these two elements has its own advantages for targeted therapy. The particles emitted by ^90^Y are more energetic and more penetrating; they are able to diffuse on a thicker layer of cells, which is an advantage for the treatment of large tumors. However, even if high energy radiation allows a more uniform irradiation of the tumor, the risk of imposing an excessive dose of radiation on the adjacent tissues is very present. For its part, the ^177^Lu emits less energetic radiation, more suited to small tumors. In addition, the energy of its γ radiation is sufficient to allow detection by scintigraphy and establish dosimetry during the therapy sequences [159].

The first analog to be studied was [^90^Y]-DOTATOC (Octreother^®^), and the first treatment sessions quickly showed good results, stopping the progression of the tumor [72,160,161]. Many studies on this long-used treatment have made it possible to observe a good tolerance for this radiotracer, with fairly mild side effects (fatigue) and in very rare cases a little more severe ones (nausea). However, it also showed some toxicity for the kidneys and the bones, these two aspects being the dose-limiting factors for the patient. In vitro, a greater affinity for SSTR2 has been demonstrated for [^90^Y]-DOTATATE compared to [^90^Y]-DOTATOC [64]. However, for the diagnosis in humans, a better contrast between the kidneys and the tumor was found for [^111^In]-DOTATOC compared to [^111^In]-DOTATATE [73], which may explain the wider use of DOTATOC analog. Despite this, these two analogs have relatively similar properties and have proven to be effective treatment methods that improve survival in some patients with neuroendocrine tumors (approximately 50 months vs. 18 months without treatment) [162]. In a Phase IIA study with [^90^Y]-DOTALAN (MAURITIUS trial), this one demonstrated lower tumor uptake in neuroendocrine tumors compared to ^90^Y-DOTATOC, but could be of potential interest for other tumors, such as HCC or lung cancers [163]. With the perspective of several years of clinical use, PRRT with ^90^Y-labeled somatostatin analogs appears to be well-tolerated with favorable long-term outcome. Unfortunately, Phase III studies are still lacking [164,165].

**Figure 10:**
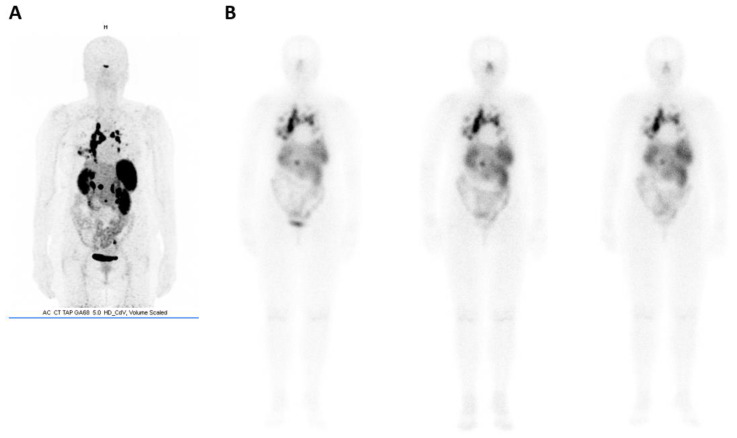
(**A**) [^68^Ga]-DOTATOC (Somakit^®^) and (**B**) [^177^Lu]-DOTATATE (Lutathera^®^, cures 1, 2, and 3) imaging of a patient treated for progressive metastatic midgut NET (images courtesy of Centre Eugene Marquis, Rennes, France).

The same analogs have also been radiolabeled with lutetium-177. Initially, [^177^Lu]-DOTATOC was used in cases of relapse of neuroendocrine tumors after treatment with [^90^Y]-DOTATOC. Despite satisfactory results [166], its subsequently developed analog [^177^Lu]-DOTATATE has shown more promise, mainly due to a more significant retention time in the tumor. For this reason, octreotate analog (TATE) is being preferred to octreotide (TOC) for labeling with lutetium [164,167]. It is also important to note that, unlike ^90^Y, no cases of nephrotoxicity after treatment with ^177^Lu have been reported. In 2005, the possibility of combining these two β^−^ emitters for therapy in cases where tumors of variable sizes are detected, was demonstrated [168]. From there, different treatment combinations between the four main systems ([^90^Y]-DOTATOC, [^90^Y]-DOTATATE, [^177^Lu]-DOTATOC, and [^177^Lu]-DOTATATE) have proven to be interesting and sometimes even more effective than using a single treatment modality [169,170]. Similarly, combination treatments with non-labeled somatostatin analogs, chemotherapy, targeted therapy, and/or radiosensitizers might further improve the efficacy and/or tolerability [171,172]. [^177^Lu]-DOTATATE has been investigated in a phase III trial, in well-differentiated, unresectable or metastatic, progressive midgut neuroendocrine tumors (Netter 1 trial). Treatment with [^177^Lu]-DOTATATE resulted in a significant tumor response rate of 18% compared with 3% in the high-dose octreotide LAR group, coupled with a 79% risk reduction for disease progression or death [173]. Following these positive findings, [^177^Lu]-DOTATATE was granted marketing authorization in this indication, both in Europe and in the US (Lutathera^®^) [174]. Coupled with ^68^Ga-imaging (Figure 10), it represents a powerful theranostic tool for the management of neuroendocrine tumors (NETs) [175]. Current research with [^177^Lu]-DOTATATE aims to improve the safety and efficacy of this procedure, enlarge possible indication, notably in advanced, poorly-differentiated, GEP-NETs, [176,177] or other NETs, such as pheomochromocytoma or paraganglioma [178,179].

#### 3.2.2. Rhenium-188 and Other β-Emitting Radionuclides

**Figure 11:**
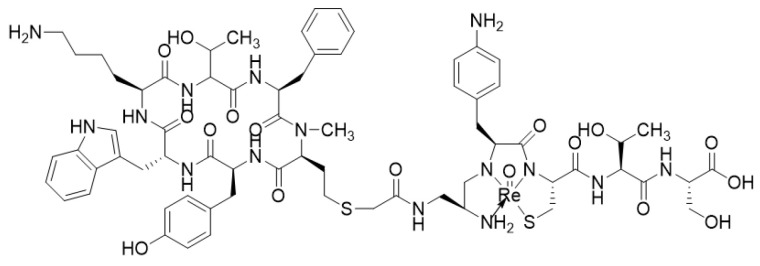
[^188^Re]-P2045.

Despite equally interesting characteristics, rhenium-188 remains widely less used than ^90^Y and ^177^Lu [180]. This is mainly due to more difficult chemistry and the unavailability of a pharmaceutical-grade ^188^W/^188^Re generator, as compared to the other two. Vapreotide and Lanreotide analogs have been described in the literature with ^188^Re. They have been investigated in experimental cancer models (e.g., pancreas, colorectal, lungs and cervical) to reduce tumor growth [181,182,183,184]. [^188^Re]-Lanreotide notably demonstrated favorable pharmacokinetics and distribution profiles (tumor-to-liver ratio) in HCC-bearing rats compared to healthy ones [185]. Another example is an equivalent to Depreotide (P829). After the development of ^99m^Tc-Depreotide for imaging, the idea was to label this compound with ^188^Re, to assess its potential in vivo. Although the radiolabeling proceeded successfully, the study showed unacceptable toxicity to non-target organs. To improve its properties, structural modifications of the peptide sequences close to the chelating moiety were tested. This optimization led to P2045, which showed better accumulation in the tumor, weaker retention in the kidneys, and faster urinary excretion than [^99m^Tc]-depreotide [186]. This new rhenium-based analog of depreotide, [^188^Re]-P2045 (Figure 11), went up to phase I in therapy for small cell lung cancer [187] and has shown promising in vivo results in the treatment of pancreatic tumors in mice [188]. To the best of our knowledge, no HYNIC-TOC/TATE or demotate derivatives have yet been radiolabeled with rhenium. Recent research with rhenium isotopes has been focusing on tricarbonyl core derivatives for the labeling of NOTA-SSAs [96].

In a theranostic perspective, other β-emitting nuclides could have a potential interest—such as ^47^Sc (T_1/2_ = 3.35 d, E_βmax_ = 600.8 keV), ^67^Cu (T_1/2_ = 2.58 d, E_βmax_ = 577 keV), and ^161^Tb (T_1/2_ = 6.91 d, E_βmax_ = 593 keV)—to be coupled with ^44^Sc, ^64^Cu, and ^152^Tb/^155^Tb respectively [129,158,189]. To date, no ^67^Cu-labeled somatostatin analogs have been described so far, and only very preliminary studies have been described with [^161^Tb]-DTPA-Octreotide and [^47^Sc]-DOTATOC [190,191].

#### 3.2.3. Alpha and Auger Emitters

Recently, alpha emitters have attracted particular attention for radionuclide therapy. Long confined to hematological tumors, they are now being considered for the potential treatment of solid tumors [192]. In vitro, α-labeled somatostatin analogs (DOTATOC and DOTATATE) demonstrated a significantly higher killing effect compared to ^177^Lu [193,194,195]. [^213^Bi]- and [^225^Ac]-labeled DOTATOC (^213^Bi: T_1/2_ = 45.6 min, E_α_ = 5.88 MeV; ^225^Ac: T_1/2_ = 9.92 d, E_α_ = 5.83 MeV) have demonstrated promising therapeutic effects in pre-clinical animal studies [196,197]; whereas [^213^Bi]-DOTATATE, investigated in human small cell lung carcinoma and rat pancreatic tumor models, demonstrated a great therapeutic effect in both small (50 mm^3^) and large (200 mm^3^) tumors, but with a higher probability for stable disease in small tumors [198]. First, and, to date, the only clinical experience with [^213^Bi]-DOTATOC, was published by Kratochwil et al., and included seven patients with advanced NETs with liver metastases refractory to treatment with [^90^Y]-DOTATOC or [^177^Lu]-DOTATOC [199]. It demonstrated specific tumor binding, lower toxicity than with β-irradiation and partial remission of metastases. Two years after intra-arterial injection of [^213^Bi]-DOTATOC, all seven patients were still alive. Regarding ^225^Ac, a first-in-human study included 10 patients with progressive NETs after β-PRRT. As with ^213^Bi, [^225^Ac]-DOTATOC was well tolerated and effective [200]. A recent study with [^225^Ac]-DOTATATE confirmed the potential of these radiotracers as an additional, and valuable, treatment option for patients who are refractory to [^177^Lu]-DOTATATE therapy. 32 patients with previous [^177^Lu]-DOTATATE therapy were treated with [^225^Ac]-DOTATATE (100 kBq/kg body weight). The response was assessed in 24 patients, with 9 stabilized diseases and 15 partial remissions [201].

**Figure 12:**
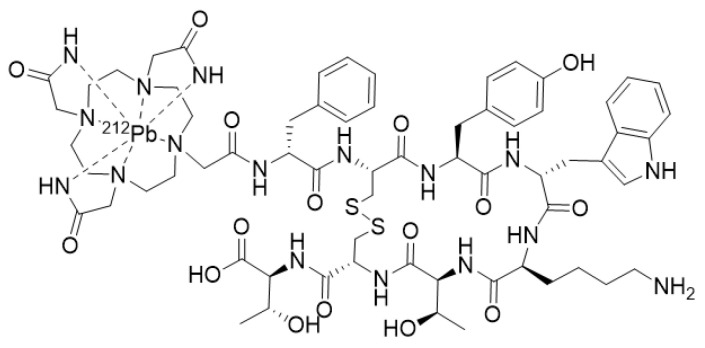
[^212^Pb]-DOTAMTATE.

Though not stricto sensu an α-emitter, lead-212 (T_1/2_ = 10.6 h) eventually decays to stable 2^08^Pb through a cascade chain with two α-emissions of potential therapeutic interest. A somatostatin analog, DOTAMTATE (Figure 12), has been labeled with ^212^Pb and investigated in a murine model of neuroendocrine tumor. Results showed a promising safety index with a 3.2-fold increase in median survival and one-third of the animals being tumor-free. A combination with 5-FU (Fluorouracyl) was able to durably cure approximately 80% of the animals. [202] Given these promising outcomes, a Phase I dose-escalation clinical trial has recently been started with [^212^Pb]-DOTAMTATE (AlphaMedix™) including 50 patients with unresectable or metastatic neuroendocrine tumors (NCT03466216). Preliminary results (nine patients enrolled) demonstrated a favorable safety profile at the tested doses [203].

Cyclotron-produced astatine-211 (T_1/2_ = 7.2 h, E_α_ = 5.87 MeV) is another very promising α-emitting radionuclide. Astatine is the heaviest halogen with a behavior somehow similar to iodine, but, in certain circumstances, it also displays significant metallic characteristics [204]. Direct astatination of somatostatin analogs is feasible, through tyrosine residues, but it led to poor stability of the resulting analogs, therefore different prosthetic groups have been developed [205,206,207]. Although *N*-(3-[^211^At]astato-4-guanidinomethylbenzoyl)-Phe^1^-octreotate ([^211^At]-AGMBO) and *N^α^*-(1-deoxy-d-fructosyl)-*N^ε^*-(3-[^211^At]astatobenzoyl)-Lys^0^-octreotate ([^211^At]-GABLO) showed disappointing biodistribution results, with poor tumor uptake, [^211^At]-SPC-octreotide displayed a more favorable biodistribution profile, and a dose-dependent apoptosis in an NSCLC murine model.

Auger electron emitters are also very potent for specific tumor cell killing, sparing surrounding cells, with a highly localized energy deposition. Indium-111 emits Auger electrons (E_Ae-_ = 19 keV, 16%), and, as such, has been investigated for therapy. Several clinical trials have been undertaken with high doses of [^111^In]-Pentetreotide. A first study with 20 patients that had neuroendocrine progressive tumors demonstrated stabilization of the disease in 5 patients, and tumor shrinkage in 5 others. All of them had received a cumulated dose higher than 20 GBq [208]. In a study with 50 SSTR-positive patients treated with cumulated doses from 20 to 160 GBq, of which 40 were evaluable, there was a stabilization in 14 patients, minor remission in 6 and partial remission in 1, with mild bone marrow toxicity [209]. However, half of the patients receiving more than 100 GBq developed a myelodysplastic syndrome or leukemia. A dose of 100 GBq was thus considered the maximal tolerated activity. Another study with 27 patients with GEP-NETs found that two doses of 6.6 GBq (180 mCi) were safe and well-tolerated, demonstrating a clinical benefit in 62% of patients [210]. Benefit of ^111^In-Pentetreotide treatment was shown to last at least 6 months for 70% of patients, while only 31% of them still had sustained benefit after 18 months [211]. Efficacy in large tumors and end-stage patients is limited, mainly because of heterogeneous radiopharmaceutical uptake due to poor tumor vascularity and central necrosis [212]. This has been demonstrated by Capello et al. in a rat tumor model, with different sizes of tumors [213]. Effects were much more pronounced in small (≤ 1 cm^2^) tumors than in large (≥8 cm^2^). They also found a significant increase in tumor receptor density after tumor regrowth, indicating repeated injections would probably be more efficient than single-dose treatment. It could also be worth using PRRT with Auger emitters in an adjuvant setting after surgery, to destroy occult metastases. A final example is [^58m^Co]-DOTATOC. This radiotracer presented for potential use in Auger-based therapy, particularly for disseminated tumor cells and micrometastases, appears to have more beneficial in vitro properties than those of [^177^Lu]-DOTATATE, with a significantly more efficient cell killing effect per cumulated decay, which has to be confirmed in vivo [127].

## 4. Antagonists vs. Agonists

Pharmacomodulation around the synthetic somatostatin analogs has led to a change of chirality in the first amino-acid (from d to l form) and in cysteine number 2 (from l to d form). These modifications have given a new class of SSTR specific compounds with antagonist effects (Table 4). From a pharmacological point of view, the biological and molecular mechanisms responsible for their targeting effectiveness in vivo are completely different. After binding to an SST receptor, an agonist analog is internalized into the cell as a ligand-receptor complex. This internalization allows it to accumulate in the cell, and to increase the amount of radiation emitted. This very powerful and specific internalization mechanism enables efficient in vivo targeting of receptors. This phenomenon does not occur (or very little) for somatostatin antagonists, and they do not stimulate the G-protein coupled to the SSTR with an associate blockage of the agonist-induced activity. Surprisingly, it has been shown that targeting receptors can also be effective without internalization of the ligand-receptor complex, and some antagonist analogs can sometimes behave better than agonists (e.g., better accumulation in tumor, poor kidney retention, and rapid clearance) [214,215]. This high tumor uptake appears to be a consequence of a greater number of target binding sites for antagonists and a more slowly dissociation than for agonists, which allows for a longer accumulation of radiation [216,217]. The hypothesis of a ligand rebinding mechanism has been put forward, but this still requires some investigation before it can be validated. These first results were confirmed by preclinical studies and by preliminary clinical trials and seems to show superior results for antagonist-based tracers than agonists [218,219,220,221]. The first comparative study of antagonists with Octreoscan^®^ confirmed the good characteristics of the [^111^In]-DOTA-BASS analog, and better accumulation at the level of the tumor and better visualization of metastases. It was truly the first proof of the concept of antagonist SSTRs imaging [222].

Concerning the affinity for each SSTR subtype, it turned out that the nature of the chelator and the radiometal is of great importance for the in vivo pharmacokinetic fate (mainly for the tumor uptake and retention time) [223]. Ultimately, copper-64 based radiotracers seem to be more interesting, especially when comparing their contrast ratio between the tumor and normal tissues which increases over time—a direct consequence of their higher half-life. The influence of radiometals (^111^In, ^90^Y, ^177^Lu, ^64^Cu, and ^68^Ga) and chelates (DOTA and NODAGA) on three antagonist families (LM3, JR10, and JR11) were also studied. On the radiometric side, the overall affinity of [^68^Ga]-DOTA was found to be much lower than for the other elements, which is the opposite of the results obtained with the agonists. For the chelate, the substitution of DOTA by NODAGA seems to greatly improve the affinity of the antagonist analogs. During this study, two particularly promising platforms emerged, DOTA-JR11 and NODAGA-JR11 [224]. Another example highlighting the influence of the chelate is 406-040-15 (cyclo (2–11) H-Cpa-DCys-Asn-Phe-Phe-DTrp-Lys-Thr-Phe-Thr-Cys-2NalNH_2_), a pansomatostatin analog, with an SSTR3 antagonist behavior. Chelation to DOTA turned this analog to an agonist [225]. Note that the first antagonist labeled via a [^99m^Tc]-tricarbonyl core has been described. ^99m^TcL-sst2-ANT (with L = tridentate ligand type N, S, N) has shown very promising in vivo behavior, but requires some modifications to improve its pharmacokinetics [226].

**Figure 13:**
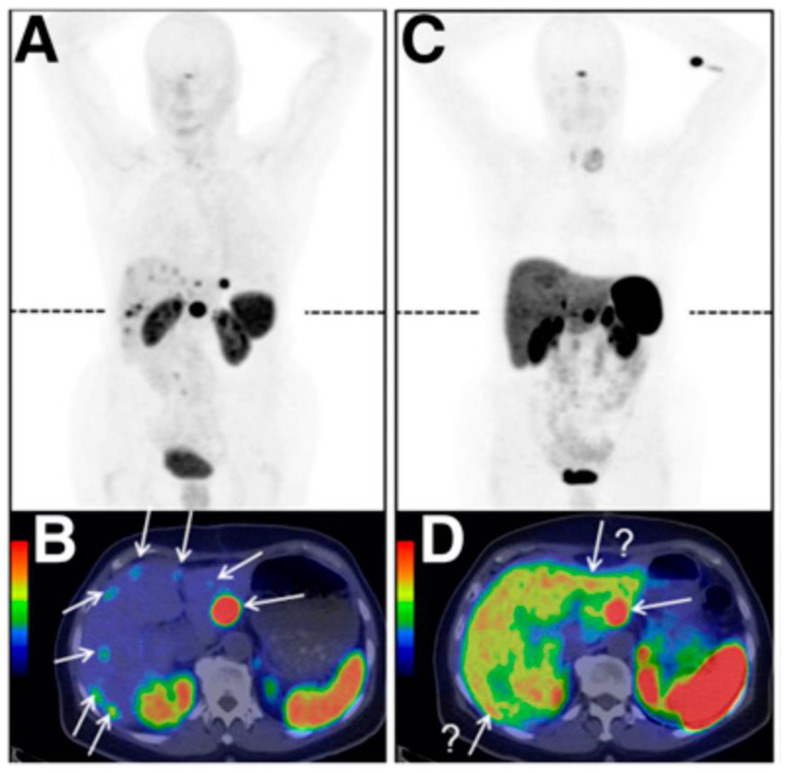
Comparison between [^68^Ga]-OPS202 (**A**,**B**) and [^68^Ga]-DOTATOC (**C**,**D**) PET/CT images of the same patient with ileal neuroendocrine tumours, showing bilobar liver metastases (from Rangger et al. [233]).

As for imaging, antagonists are also an interesting alternative for therapy. As discussed above, the first proof of the feasibility of imaging using antagonists was highlighted by comparing Octreoscan^®^ and [^111^In]-DOTA-BASS. However, this analog has shown only a very modest affinity for the SSTR2 receptor subtype targeted in the therapy of neuroendocrine tumors [214]. To overcome this problem, the second generation of somatostatin antagonists was synthesized to improve affinity for this receptor. DOTA-JR11 showed the highest affinity for SSTR2 and was selected for use in targeted therapy [218]. A pilot study to assess the possibility of treatment with [^177^Lu]-DOTA-JR11, by comparing it to [^177^Lu]-DOTATATE, was carried out. This new antagonist has shown favorable properties, such as better accumulation in the tumor and a higher dose received by the tumor, thanks to a longer retention time [227]. Further developments led to a theranostic pair with JR11: one with a NODAGA chelator (satoreotide trizoxetan, OPS-202) and one with DOTA chelator (satoreotide tetraxetan, OPS-201) [228,229]. Satoreotide trizoxetan is currently radiolabeled with ^68^Ga and used in PET imaging clinical trials (Figure 13) [230,231]. Satoreotide tetraxetan radiolabeled with ^177^Lu has been evaluated in a therapeutic clinical trial [232]. First clinical results for this somatostatin antagonist theranostic pair seem to be promising with high sensitivity for neuroendocrine tumors and require further studies in larger patient population.

## 5. Future Prospects

Regarding clinically established somatostatin analogs, the development of kit-based ^68^Ga radiotracers, as well as cyclotron production of gallium-68 should improve their availability and worldwide dissemination. Further clinical translation of ^64^Cu- and ^18^F-based somatostatin SSAs could also represent an attractive alternative. For therapy, current research focuses on optimizing the dose received by the tumor while sparing healthy tissues. Fractionation, as well as combination of ^90^Y and ^177^Lu, have demonstrated their interest [168,234]. The same approach with other treatment modalities, such as external-beam radiotherapy or chemotherapy could enhance treatment response [235,236]. Targeted α-therapy also seems to hold promises and is currently attracting much interest, notably from the industry.

Recent developments showed a switch from agonist to antagonist derivatives, demonstrating higher efficacy. With the advent of new promising radionuclides and somatostatin analogs with better pharmacokinetic properties and binding profiles, the future looks bright for radiolabeled somatostatin analogs, expanding their use for wider indications, than just GEP-NETs. With peptide derivatives with improved targeting, tumors with lower SSTR expression might nonetheless be clinically relevant. In this context, as already demonstrated with some analogs, use of somatostatin-based radiopharmaceuticals might be of interest in pulmonary or hepatic cancers, warranting further studies. The development of bivalent radiotracers to target several receptors concomitantly expressed could be of interest to improve targeting [237]. Similarly, improved detection and sensitivity could be achieved using bimodal agents [238]. Besides, the clinical success for radiolabeled somatostatin analogs both with diagnostic and therapeutic radionuclides paved the way for new promising peptide derivatives, such as bombesin, neurotensin, or CXCR4 ligands, and, in a similar way, PSMA ligands, for cancer theranostics [49,233,239,240].

## Figures and Tables

**Table 1:** SSTRs expression in different tumor types.

Tumor Type	SSTR Expression	Ref
Astrocytoma	+	[17]
Breast carcinoma	+ (SSTR2)	[11]
Cholangiocarcinoma	+ (SSTR2)	[18]
Colorectal carcinoma	-	[17]
Endometrial carcinoma	-	[17]
Ependymoma	+ (SSTR1, SSTR5)	[11]
Esophageal carcinoma	-	[17]
Ewing sarcoma	-	[17]
Exocrine pancreatic tumor	-	[17]
Gastric carcinoma	+ (SSTR1 > SSTR2, SSTR5)	[11]
Gastrinoma	**+** (SSTR2)	[17]
Glioblastoma	-	[17]
Growth hormone-producing pituitary adenoma	**+** (SSTR2, SSTR5)	[17]
Gut carcinoid	**+** (SSTR2 > SSTR1, SSTR5)	[17]
Hepatocellular carcinoma	+ (SSTR2, SSTR5)	[19]
Insulinoma	+ (SSTR1, SSTR2, SSTR3)	[20]
Leiomyoma	+	[17]
Lymphoma	+ (SSTR2)	[11]
Medullary thyroid carcinoma	+ (SSTR2)	[11]
Medulloblastoma	**+** (SSTR2)	[17]
Meningioma	**+** (SSTR2)	[17]
Neuroblastoma	**+** (SSTR2)	[17]
Non-functioning pituitary adenoma	+ (SSTR3 > SSTR2)	[17]
Non-small cell lung cancer	-	[17]
Ovarian carcinoma	+	[17]
Paraganglioma	**+** (SSTR2)	[17]
Pheochromocytoma	**+** (SSTR1, SSTR2)	[17]
Prostate carcinoma	+ (SSTR1)	[17]
Renal cell carcinoma	+ (SSTR2)	[11]
Small cell lung cancer	+ (SSTR2)	[17]
Urinary bladder carcinoma	-	[17]

Bold +, receptors with particularly high density and incidence. Subtypes preferentially expressed are listed in parentheses, only when compelling evidence is available (immunohistochemistry or autoradiography). Adapted from [11] and [17].

**Table 2:** Some of the main radionuclides studied for imaging and therapy (SPECT—Single-Photon Emission Computed Tomography; PET—Positron Emission Tomography).

Radionuclide	Half-Life (h)	Type of Emission	Energy of Emitted Radiation (keV)	Source	Application
^99m^Tc	6.01	γ	140	Generator	SPECT imaging
^111^In	67.4	γ	172, 245	Cyclotron	SPECT imaging
^18^F	1.83	β^+^	634	Cyclotron	PET imaging
^64^Cu	12.7	β^+^/γ/β^-^	653	Cyclotron	PET imaging
^68^Ga	1.1	β^+^	1190	Generator/Cyclotron	PET imaging
^90^Y	64.1	β^−^	2284	Generator	Therapy
^177^Lu	160.8	β^−^/γ	497	Cyclotron	Therapy
^188^Re	17	β^−^/γ	2118	Generator	Therapy
^211^At	7.2	α	5870	Cyclotron	Therapy
^225^Ac	238	α	5830	Generator	Therapy

**Table 3:** Peptidic sequences of the main somatostatin agonist analogs. Differences towards Octreotide (OC) are highlighted in red.

Peptide	Peptidic Sequence
OC Octreotide	d-Phe^1^-cyclo(Cys^2^-Phe^3^-d-Trp^4^-Lys^5^-Thr^6^-Cys^7^)Thr(ol)^8^
LAN Lanreotide	β-d-Nal^1^-cyclo(Cys^2^-Tyr^3^-d-Trp^4^-Lys^5^-Val^6^-Cys^7^)Thr^8^-NH_2_
VAP Vapreotide	d-Phe^1^-cyclo(Cys^2^-Phe^3^-d-Trp^4^-Lys^5^-Val^6^-Cys^7^)Trp^8^-NH_2_
TOC [Tyr^3^]-Octreotide	d-Phe^1^-cyclo(Cys^2^-Tyr^3^-d-Trp^4^-Lys^5^-Thr^6^-Cys^7^)Thr(ol)^8^
TATE [Tyr^3^]-Octreotate	d-Phe^1^-cyclo(Cys^2^-Tyr^3^-d-Trp^4^-Lys^5^-Thr^6^-Cys^7^)Thr^8^
NOC [1-Nal^3^]-Octreotide	d-Phe^1^-cyclo(Cys^2^-1-Nal^3^-d-Trp^4^-Lys^5^-Thr^6^-Cys^7^)Thr(ol)^8^
NOC-ATE [1-Nal^3^, Thr^8^]-Octreotide	d-Phe^1^-cyclo(Cys^2^-1-Nal^3^-d-Trp^4^-Lys^5^-Thr^6^-Cys^7^)Thr^8^
BOC [BzThi^3^]-Octreotide	d-Phe^1^-cyclo(Cys^2^-BzThi^3^-d-Trp^4^-Lys^5^-Thr^6^-Cys^7^)Thr(ol)^8^
BOC-ATE [BzThi^3^, Thr^8^]-Octreotide	d-Phe^1^-cyclo(Cys^2^-BzThi^3^-d-Trp^4^-Lys^5^-Thr^6^-Cys^7^)Thr^8^

**Table 4:** Main somatostatin antagonist analogs. Differences towards octreotide (OC) are highlighted in red.

*Antagonist Peptide*	*Peptidic Sequence*
Sst2-ANT (BASS)	p-NO_2_-Phe^1^-cyclo(d-Cys^2^-Tyr^3^-d-Trp^4^-Lys^5^-Thr^6^-Cys^7^)d-Tyr^8^-NH_2_
LM3	p-Cl-Phe^1^-cyclo(d-Cys^2^-Tyr^3^-d-Aph^4^(Cbm)-Lys^5^-Thr^6^-Cys^7^)d-Tyr^8^-NH_2_
JR10	p-NO_2_-Phe^1^-cyclo(d-Cys^2^-Tyr^3^-d-Aph^4^(Cbm)-Lys^5^-Thr^6^-Cys^7^)d-Tyr^8^-NH_2_
JR11 (Satoreotide)	p-Cl-Phe^1^-cyclo(d-Cys^2^-Aph^3^(Hor)-d-Aph^4^(Cbm)-Lys^5^-Thr^6^-Cys^7^)d-Tyr^8^-NH_2_
